# Xiphoidectomy: A Surgical Intervention for an Underdocumented Disorder

**DOI:** 10.1155/2016/9306262

**Published:** 2016-11-09

**Authors:** Dirk Pieter Hogerzeil, Klaas Albert Hartholt, Mark Rem de Vries

**Affiliations:** Department of Surgery-Traumatology, Reinier de Graaf Groep, Postbus 5011, 2600GA Delft, Netherlands

## Abstract

Two patients who presented with nonspecific thoracic and upper abdominal symptoms and tenderness of the xiphoid process are discussed. Both patients had undergone extensive examinations, but no source for their symptoms could be found. Plain chest radiographs revealed an anterior displacement of the xiphoid process in both patients. Physical examination confirmed this to be the primary source of discomfort. Anterior displacement of the xiphoid process may be the result of significant weight gain. Repeated trauma of the afflicted area, unaccustomed heavy lifting, exercise, and perichondritis are, amongst other causes, believed to contribute to the development of xiphodynia. Both patients were treated by performing a xiphoidectomy, resulting in disappearance of the symptoms.

## 1. Introduction

Xiphodynia was first reported in 1712. The affliction can present itself in many ways, including chest or abdominal pain [1]. Since Lipkin et al. reported 4 cases of xiphodynia in 1955, only a handful of publications on this topic have followed. Since then, the ailment has been given many names; the most common ones are xiphodynia, xiphoid syndrome and xiphoidalgia, amongst others.

Over the years many possible causes for xiphodynia have been suggested in the literature, although still very little is known about the etiology [2–4]. Some publications suggest that inflammation due to (repeated) mechanical injury might contribute to the cause [2]. Others suggest that anterior displacement of the xiphisternal angle is a cause for prominence of the xiphoid process and therefore prone to mechanical injury and subsequent inflammation [3]. Lastly, some authors suggest repeated (micro)traumas to the xiphoid process, such as occupational injuries, acceleration and deceleration injuries, and sports related injuries as the main cause in the etiology of xiphodynia [4, 5].

One of many possible reasons for xiphodynia to be an unknown disorder is the wide variety of symptoms it may produce. For example, xiphodynia could mimic serious disorders, for example, abdominal or cardiac disease [4]. Furthermore, the xiphoid process is often not included in routine physical examination of patients and might therefore be easily missed.

Treatments thus far have included ultrasound therapy, injection of local anesthetic agents, low-level laser therapy (LLLT), and topical anti-inflammatory gel. Xiphoidectomy as a therapy has only scarcely been reported [4, 5]. Two cases of xiphodynia that presented to our clinic, both treated with xiphoidectomy, are shown.

## 2. Case Presentation

### 2.1. Case 1

**Figure 1:**
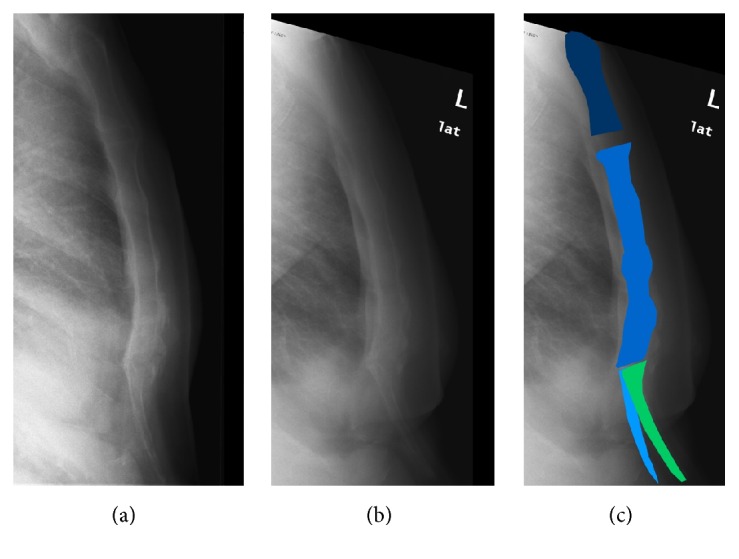
Progression of the anterior displacement of the xiphoid process.

A 61-year-old male presented with complaints of nausea, abdominal pain, tenderness of the xiphoid process, and the feeling of “luxation” of the sternum, as if bones were sliding over each other as he describes. He quit smoking a few years earlier, which resulted in significant weight gain. The patient was an overweight mechanic (BMI 31.7 kg/m^2^), who worked on buses for which he often had to carry large tires. His current complaints however prevented him from being able to work. The symptoms were thought to be gastrooesophageal reflux, and the patient was analysed by a gastroenterologist who started treatment with a proton pump inhibitor. Upper gastrointestinal endoscopy revealed, apart from a small sliding diaphragmatic hernia (3 cm), no abnormalities. Cardiac pathology was ruled out by a cardiologist. Previously taken plain radiographs of the sternum before the weight gain showed a slight anterior displacement of the xiphoid process (Figure 1(a)). At three years after the onset of his complaints a second radiograph of the sternum was obtained, which showed progression of the anterior displacement of the xiphoid process (Figure 1(b)). This progression (Figure 1(c)) was thought to be due to the patients' weight gain, causing anterior displacement as a result of pressure on the xiphoid process by the panniculus. A xiphoidectomy was performed. Postoperatively the patient experienced no more pain, and he could return to work. During eighteen months of follow-up, the patient reported none of the symptoms he complained of before.

### 2.2. Case 2

**Figure 2:**
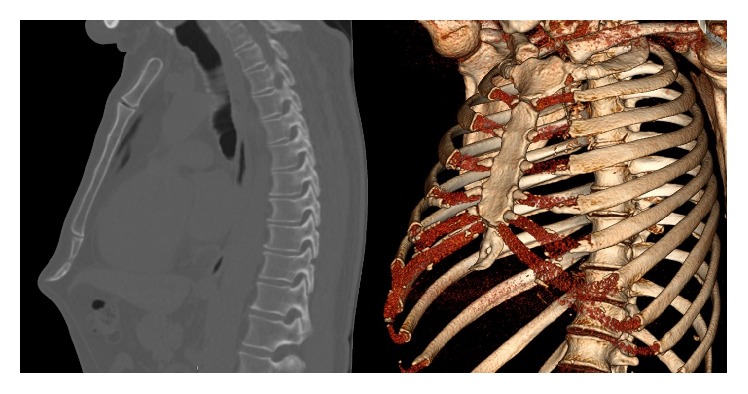
Computerized tomography of xiphoid process protrusion.

A 55-year-old male was referred to our clinic by his General Practitioner regarding complaints of his xiphoid process. Apart from routine physical examination no further analysis had been performed by the general practitioner. The patient had been overweight (BMI 30.6 kg/m^2^) but had recently, in an effort to live a healthier life, lost 17 kg of bodyweight, which resulted in a BMI of 25.6 kg/m^2^. However, since the weight loss the patient experienced a pain sensation of the xiphoid process. In his daily life, he works as a mechanic who operates heavy machinery and lifts heavy items. Ever since his weight loss he noticed that his xiphoid process was protruding and lifting items started to get bothersome and painful. In this case a computerized tomography (CT) was available (Figure 2). This chest CT was made in the process of analysing his symptoms. The images show a protruding xyphoid process. A xiphoidectomy was performed. Postoperatively the patient reported no more pain and remained symptom-free during thirty months of follow-up.

## 3. Discussion

The aetiology of xiphodynia is still not clear. Multiple factors are believed to have at least a role in the development of xiphodynia. Some of these factors are perichondritis, osteochondritis, heterotopic ossification, resuming heavy work after a period of inactivity, (repeated) trauma, exercise, and unaccustomed heavy lifting [1, 5, 6]. Far too often little attention is paid to the xiphoid process during physical examination resulting in patients having these bothersome symptoms for years.

However, caution must be exercised in diagnosing xiphodynia for symptoms may be secondary to cardiac, abdominal, or any other thoracic disease or could be referred pain [4].

In general xiphodynia can be diagnosed by reproducing the symptoms familiar to the patient, by applying light pressure on the xiphoid process during physical examination. As mentioned earlier, a wide variety of factors may contribute to the etiology of xiphodynia. Our theory however, as represented by the two cases, is that a period of overweight, and the hereby inferred distension of the abdomen, causes mechanical traction on the xiphoid process by means of the attaching muscles and thus, over time, causing anterior displacement of the xiphoid process. Then, after the patient has lost most of the excess weight, the abdomen returns to its original state leaving the xiphoid process displaced anteriorly. This in term may then contribute to repeated (micro)traumas in everyday labour, sports, and other activities. Also the anterior displacement in itself causes discomfort to the patient. Anterior displacement may also occur during or after pregnancy in which, too, distension of the abdomen may cause traction on the xiphoid process. We suggest that substantial weight gain contributed to an anterior displacement of the xiphoid process in both our cases. The subsequent protrusion of the xiphoid process then results in repeated traumas, irritation, and inflammation and therefore causes our patients' xiphodynia.

Many other treatment options have been suggested, such as combined anesthetic and corticosteroid injections, LLLT, and topical anti-inflammatory gel. It is our belief however that only xiphoidectomy offers a definitive solution to the issue in these specific cases.

A number of anatomical varieties of the xiphoid process exist. However, the innervation and attachment of abdominal structures remain the same. A small part of the m. rectus abdominis and the anterior costoxiphoid ligament are attached anteriorly, and the posterior costoxiphoid ligament, some fibers of the diaphragm, and the transverse thoracis muscle are attached posteriorly. The aponeuroses of the external oblique, the internal oblique, and the transverse abdominis muscle are attached laterally [7].

The first observation of disorders of the xiphoid process dates back to 1712; however to date only a handful of papers have been published on the issue and it is our hope that more future studies will report on the matter and shed light on the etiology, prevalence, incidence, and optimal treatment [1, 3].

## References

[1] Lipkin M., Fulton L. A., Wolfson E. A. (1955). The syndrome of the hypersensitive xiphoid. *The New England Journal of Medicine*.

[2] Yapici Ugurlar O., Ugurlar M., Ozel A., Erturk S. M. (2014). Xiphoid syndrome: an uncommon occupational disorder. *Occupational Medicine*.

[3] Maigne J.-Y., Vareli M., Rousset P., Cornelis P. (2010). Xiphodynia and prominence of the xyphoid process. Value of xiphosternal angle measurement: three case reports. *Joint Bone Spine*.

[4] Simpson J. K., Hawken E. (2007). Xiphodynia: a diagnostic conundrum. *Chiropractic and Osteopathy*.

[5] Howell J. M. (1992). Xiphodynia: a report of three cases. *Journal of Emergency Medicine*.

[6] Migliore M., Signorelli M. (2012). Episodic abdominal and chest pain in a young adult. *Journal of the American Medical Association*.

[7] Gray H., Lewis W. H. (1918). *Anatomy of the Human Body*.

